# Analysis of Toxigenic *Fusarium* Species Associated with Wheat Grain from Three Regions of Russia: Volga, Ural, and West Siberia

**DOI:** 10.3390/toxins11050252

**Published:** 2019-05-05

**Authors:** Tatiana Gagkaeva, Olga Gavrilova, Aleksandra Orina, Yuri Lebedin, Ilya Shanin, Pavel Petukhov, Sergei Eremin

**Affiliations:** 1 Laboratory of Mycology and Phytopathology, All-Russian Institute of Plant Protection, St. Petersburg, 196608 Pushkin, Russia; olgavrilova1@yandex.ru (O.G.); orina-alex@yandex.ru (A.O.); 2 The Institute of Environmental and Agricultural Biology (X-BIO), University of Tyumen, 625003 Tyumen, Russia; 3 XEMA Company Limited, 105264 Moscow, Russia; info@xema-medica.com (Y.L.); info@xema-medica.com (I.S.); info@xema-medica.com (P.P.); 4 Chemical Department, M.V. Lomonosov Moscow State University, 119991 Moscow, Russia; eremin_sergei@hotmail.com

**Keywords:** *Fusarium* fungi, Russia, wheat grains, DNA, biomass, mycotoxins, qPCR, immunoassay, HPLC-MS/MS

## Abstract

Wheat grains collected in three regions of Russia—Volga, Ural, and West Siberia—were analyzed for triangulation of methods in analysis of toxigenic *Fusarium* species. The presence of fungi and quantitative content of their biomass were detected by using various analytical methods, including a mycological and immunochemical methods, and quantitative PCR. Additionally, an enzyme-linked immunosorbent assay and high-performance liquid chromatography with tandem mass spectrometry were applied for determination of mycotoxins. Regional differences were found regarding the contamination of wheat grain by *Fusarium* fungi and their toxins. The most important observation was the detection of *F. graminearum* in the Ural and West Siberian regions, where this pathogen had not been found previously. A maximum damaged grains by *F. graminearum* and *F. sporotrichioides* was found in the grain samples from West Siberia. The DNA of *F. graminearum* was detected in 19.2% and DNA of *F. sporotrichioides* was found in 84.1% of the analyzed grain samples. The amount of *Fusarium* antigens in the grain samples from the West Siberian region was 7–8 times higher than in the grain samples from the other two regions. Significant contamination of the grain with deoxynivalenol and T-2/HT-2 toxins (maximum contents were 2239 ppb and 199 ppb, respectively) was detected in the West Siberian region.

## 1. Introduction

*Fusarium* Link is a large cosmopolitan genus of ascomycete fungi consisting of numerous species that are both saprotrophic and pathogenic to cereals. The major impacts of pathogenic species on wheat include a yield reduction as well as a reduction in the marketable quality of grains.

Some *Fusarium* species are known to produce different types of mycotoxins, which are the most deleterious of natural metabolites in terms of health effects. The ability to produce mycotoxins varies between species and also between strains of the same species, but the quantity of formed metabolites depends on environmental factors. Research on the *Fusarium* problem in cereals has demonstrated that visual symptoms, fungal incidence, and mycotoxin accumulation in grains are not always closely linked [1,2,3]. Due to the problem of *Fusarium* damaged grain (FDG), there is a need for highly accurate methods for detecting the infection of grain batches and quantitative mycotoxin contamination.

It is known that several *Fusarium* species can be found together in a grain sample and even in a single grain [4,5]. The most important mycotoxin producers in small grain cereals are *F. graminearum* Schwabe, *F. sporotrichioides* Sherb., and *F. langsethiae* Torp and Nirenberg [6,7,8]. It is well known that *F. graminearum* is the main producer of trichothecenes type B (mostly deoxynivalenol, DON), while *F. sporotrichioides* and *F. langsethiae* are associated with trichothecenes type A (mostly T-2 and HT-2 toxins) [9]. Some strains of *F. graminearum* concomitantly produce another mycotoxin, zearalenone (ZEN), which shows estrogenic activity [10]. As was noticed, the climate changes have a significant impact on stages and rates of toxigenic fungi development and can modify host-pathogen interactions, also deeply influencing the conditions for mycotoxin production [11].

In light of the ubiquity and health hazards presented by some secondary metabolites of *Fusarium* fungi, many countries have introduced mandatory or guideline levels for mycotoxins in grain and grain-based products. In the Russian Federation on the legislation regarding mycotoxins in wheat grain for direct human consumption the maximum permissible levels for T-2 toxin is 100 ppb, for DON is 700 ppb, and for ZEN is 1000 ppb [12]. Often the time interval is very short between obtaining the harvested grain and the decision about its intended purpose, subsequent technological processing, and ultimately, the cost. The need for such large-scale analysis of grain led to the development and validation of numerous analytical methods utilizing a number of techniques.

The identification of the microbiological quality of grain includes two tasks, which are usually solved using fundamentally different methods. The first is the mycological identification of pathogenic *Fusarium* fungi remaining in the grains, while the second involves the quantitative detection of the metabolites that are obviously absent in plant tissue but inherent in fungi.

Traditional mycological methods, based on the stimulation of fungi growth on a nutrient medium and subsequent microscopic analysis, help to identify the spectrum of cultivated microorganisms and determine the frequency of grain infection with a specific taxon. The standard parameter used to characterize the purity of grain is the percentage of infected grain. However, it has long been acknowledged that the established percentage of grain infection is often weakly related to the quantitative presence of pathogen biomass [13,14].

Another possibility is the characterization of infection based on the number of metabolite markers of fungi that colonize the plant. For this purpose, it is possible to use the quantitative presence of both primary (e.g., proteins, fats, carbohydrates, and nucleic acids) and secondary (e.g., antibiotics, mycotoxins, and pigments) metabolites of a particular taxon.

Quantitative PCR (qPCR), which reveals the amount of DNA of one or a group of closely related microorganisms is quite popular in plant pathological research [15,16,17]. Another indirect method for assessing the toxicity of grain is the immunochemical discovery of antigens (EIA) that are produced by fungi during their vital activity [18,19,20,21].

Many strategies have been proposed for controlling the occurrence of mycotoxins in plants [22]. However, the analysis of mycotoxins is an arduous task, as they may occur in various combinations, are produced by a single or several fungal species, and are usually present in range concentrations. Currently, researchers widely utilize high-performance liquid chromatography with tandem mass spectrometry (HPLC-MS/MS) and enzyme-linked immunosorbent assays (ELISA) [23,24,25,26,27]. Regardless of the choice of analytical method, it should enable the rapid and sensitive detection of mycotoxins below the legally imposed limits. Moreover, it should be cost-effective and easy to use.

The aim of this study was to reveal the accuracy and precision of the information about *F. graminearum* and *F. sporotrichioides* infection and contamination by their mycotoxins of wheat grains from three regions of Russia using various analytical methods.

## 2. Results

The wheat grain samples were collected from three regions of Russia where, at present, information about grain infection and mycotoxin contamination of grown small grain cereals is limited in comparison to the southern and central parts of European Russia, which feature intense cereal production. It was first research of grain samples with using not only mycological method but additionally with the set of various analytical methods like as immunochemical methods, quantitative PCR and HPLC-MS/MS. A practical problem arises because a high proportion of a too-frequent zero value exists in data, in which case transformation to normality cannot be achieved. Therefore, it was used the other approaches not requiring a normal distribution like as the Mann-Whitney U test.

### 2.1. Detection of Grain Infection with *Fusarium* Fungi

*Fusarium* fungi were found in 47 (82.5%) of the analyzed grain samples using the mycological method. The largest number of infected grain samples was detected in the West Siberian region (96.1%), most of which were from Novosibirsk Oblast and Altai Krai, where the maximal percentage of FDG ranged from 35% to 38% (Table 1).

*F. graminearum* was only detected in the grain samples from the Ural and West Siberian regions. The eight grain samples from Tyumen Oblast, Novosibirsk Oblast, Altai Krai, and Krasnoyarsk Krai were contaminated by this pathogen, with a maximum infection rate of 10%.

The infection of grain by *F. sporotrichioides* was established in 33 wheat samples. The maximum of infection was found in the sample from Altai Krai from the West Siberian region (15%). Conversely, the smallest amount of *F. sporotrichioides* infection was found in the Volga region (on average, less than 1%).

In addition to *F. graminearum* and *F. sporotrichioides*, which produce trichothecenes, other species that are common contaminants of grains in this area were identified based on morphological characteristics: *F. avenaceum* (Fr.) Sacc., *F. incarnatum* (Desm.) Sacc., *F. equiseti* (Corda) Sacc., *F. acuminatum* Ellis & Everh., *F. poae* (Peck) Wollenw., *F. solani* (Mart.) Sacc., and *Fusarium* spp.

### 2.2. Quantification of *Fusarium* Biomass

The DNA of *F. graminearum* was only detected in the grain collected in the Ural and West Siberian regions (30–31% of samples). However, the average fungal DNA content was significantly higher in the grain samples from the West Siberian region, including several samples containing extremely high levels in Krasnoyarsk Krai (maximum *F. graminearum* DNA value was 1318 pg/ng of total DNA).

The DNA of *F. sporotrichioides* was detected in 100% of the samples from West Siberia, 92.3% of the samples from the Ural region, and 61.1% of the samples from the Volga region. The fungal DNA content in the grain varied significantly. The maximum amount of *F. sporotrichioides* DNA was 6740 pg/ng of total DNA, which was found in a grain sample from Krasnoyarsk Krai. In addition, samples containing a significant amount of *F. sporotrichioides* DNA were found in Bashkortostan, Penza Oblast, Altai Krai, and Tyumen Oblast.

The *Fusarium* biomass revealed by the immunoassays in tested grain samples varied significantly from non-detectable amount to the concentrations exceeding the range of assay calibration curve (>250 U/mL). The average biomass content of *Fusarium* fungi in the grain samples collected in the Volga and Ural regions was similar and varied from 23.9 to 25.6 U/mL (on average, 13.7–18.8 U/mL). Further, the *Fusarium* biomass content in the grain samples from the West Siberian region was 7–8 times higher than that in the grain samples obtained from the other two regions.

### 2.3. Detection of *Fusarium* Mycotoxins

The results of mycotoxin analysis obtained by ELISA showed that one sample from the sets of grain collected in both the Volga and Ural regions contained DON in amounts near 100–150 ppb (Table 2). Among the grain samples from the West Siberian region, DON was detected in six samples. The maximum amount of this mycotoxin was 2787 ppb in grain from Krasnoyarsk Krai. ZEN was only found in three samples from the West Siberia in minor amounts. ELISA detected T-2 toxin in one grain sample in each region, with a maximum amount of 74 ppb (Chelyabinsk Oblast, the Ural).

Data from the HPLC-MS/MS analysis revealed the presence of DON in three samples from the Volga region (maximum 74 ppb) and five samples from the Ural region (maximum 415 ppb). In the West Siberian region, DON was detected in 12 samples (maximum 2239 ppb). ZEN was found in three samples from the West Siberian region, ranging from 42 to 106 ppb, with the highest amount in a grain sample from the Krasnoyarsk Krai. T-2 toxin was found in four samples from both the Volga and West Siberian regions (maximum 29 ppb) and in two samples from the Ural region (maximum 66 ppb). The incidence of HT-2 toxin was higher than that of T-2 toxin. This mycotoxin was detected in seven grain samples from the Volga region and in eight samples from the Ural region (maximum 152–169 ppb). Additionally, HT-2 toxin was found in 22 samples from the West Siberian region (maximum 92 ppb).

### 2.4. Relationship between *Fusarium* Fungi Presence and Their Mycotoxins

**Figure 1:**
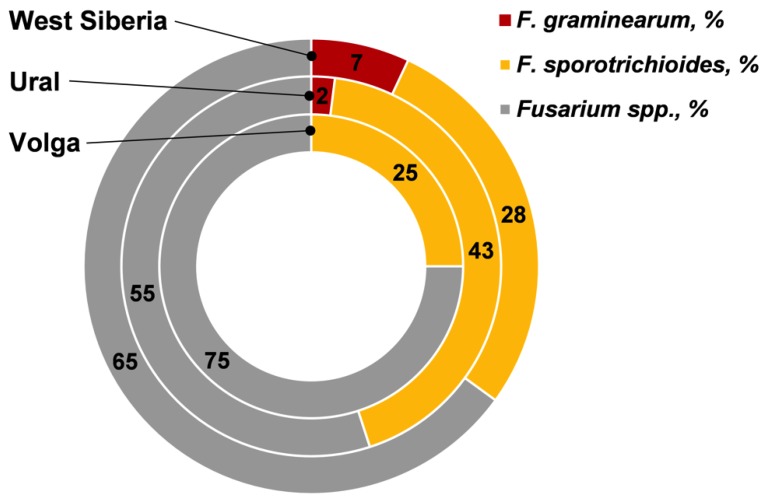
The relative proportions of *F. graminearum* and *F. sporotrichioides* in *Fusarium* fungi composition on wheat grain from the different regions of Russia.

In the *Fusarium* fungi complex detected on the grain, on average the proportion of *F. graminearum* was not high (3.6%); the proportion of *F. sporotrichioides* was significantly higher (28.2%) (Figure 1). This indicated a high correlation between FDG and *F. sporotrichioides* infection (*r* = +0.70) and the amount of DNA of this fungus (*r* = +0.38) (Table 3).

At the same time, the results of the correlation analysis revealed a significant positive relationship between the *F. graminearum* infection of grains and the fungal DNA content (*r* = +0.42) as well as total FDG (*r* = +0.43). Apparently, any presence of this aggressive pathogen leads to a significant accumulation of its biomass in grains.

The *F. graminearum* DNA content is also highly associated with DON and ZEN contents detected by ELISA and HPLC-MS/MS (*r* = +0.94–0.99). It should be noted that the mycotoxin analysis results using these two methods have showed significant similarity (*r* = +0.93–0.99).

This was confirmed by the positive correlation of the antigens of Fusarium fungi detected in the grain using the competitive EIA method with *F. graminearum* infection (r = +0.38) and with *F. graminearum* DNA content (r = +0.98).

A comparative analysis of the quantitative content of the fungal biomass using different methods (qPCR and EIA) and the results of mycological analysis showed a positive relationship between the amount of *Fusarium* antigens and *F. graminearum* infection, but this relationship was not found with *F. sporotrichioides* infection. Nevertheless, the content of *F. sporotrichioides* DNA was also positively related to the amount of *Fusarium* antigens detected by EIA (*r* = +0.56). The results demonstrate that the *Fusarium* biomass determined by using the EIA is related to the DON content (*r* = +0.96–0.97).

In our study, no relationship was established between the amount of T-2 toxin, detected by ELISA and HPLC-MS/MS, and *F. sporotrichioides* infection of grain or between the DNA content of this pathogen and the amount of *Fusarium* antigens. However, the amount of HT-2 toxin was positively related to the amount of T-2 toxin detected using both methods (*r* = +0.65–0.78).

Moreover, the amount of HT-2 toxin, as well as the sum of the T-2 and HT-2 toxins, is significantly related to the DNA content of the main producer *F. sporotrichioides* (*r* = +0.54–0.57).

## 3. Discussion

The using analytical methods provide the accuracy and correctness of the information, selection of the most effective strategies for the demonstration of existing situation with the toxin-producing fungi, which will be the platform for future researches. Despite the limited set of grain samples, we have shown the problem of *Fusarium* grain infection and mycotoxin contamination in the analyzed regions. The Ural Mountains have been considered the traditional boundary between Europe and Asia and can be a natural barrier to the spread of fungi between two parts of the continent. The vast territories extending from the Ural Mountains are characterized by contrasting climatic conditions, both territorial, and seasonal and daily. There is no doubt that a need for further studies of the quality of grain from these regions.

The consistent results obtained using the various analytical methods showed the presence of *F. graminearum* and its metabolites in wheat grains from the West Siberia (Novosibirsk Oblast, Altai Krai, and Krasnoyarsk Krai) and Ural regions (Tyumen Oblast). This is the first scientifically confirmed detection of *F. graminearum* in West Siberia. The grain samples with the highest rates of infection (10%) were also characterized by a significant presence of *F. graminearum* DNA (up to 1318 pg/ng). The *F. graminearum* infection in the grain from Krasnoyarsk Krai resulted in the accumulation of high amounts of DON more than 2–3 times the permissible level of 700 ppb (up to 2787 ppb). The mycotoxin ZEN was also found in small amounts in these samples. Despite the detection of samples containing *F. graminearum* and the high level of infection of these samples, the median value of this pathogen in the grain from all the regions was very close to zero. However, it was found the significant differences between total FDG, *F. graminearum* infection and fungal DNA revealed in grains from West Siberian region in comparison with grain from Ural and especially Volga regions.

Until now, it was believed that only isolated populations of *F. graminearum* exist in Russia, one in the Far Eastern region and others in the south and northwest parts of European Russia [28,29]. The reason for this separation was the absence of this pathogen from the plants’ mycobiota in Siberia [29,30,31,32]. The appearance of *F. graminearum* in West Siberia can be explained by various factors, including the possible introduction of the pathogen into the region with seeds or climate change [33].

It is known that rainfall and humidity during wheat flowering contribute to infection by *F. graminearum* [34]. In Table 4 provides summary analysis of temperature and precipitation for three summer months. In these regions, the flowering of spring wheat occurs in the first decade of July. The climate of Volga region is characterized by dry summer; in 2017 the amount of precipitation during three months was less than 71 mm. In the Ural and West Siberia where *F. graminearum* was detected the average rainfall for these months was 131 and 113 mm. In addition, the humidity during flowering and maturing of wheat in the Ural and West Siberia was higher than it was in the Volga region. The co-variability of measured data with observed monthly mean temperature and rainfall in the represented locations was determined. According to our data, the weather conditions only in August significantly influenced the outcome of the relationship between plants and *Fusarium* fungi. A positive relationship was established only between the amount of precipitation in August and the DNA content of *F. graminearum*, antigens and DON (*p* ≤ 0.01). Increasing temperatures in August significantly reduced the total FDG, infection rate, and DNA content of *F. sporotrichioides* (*p* ≤ 0.01).

Previously it was shown that *F. graminearum* population dynamics have been influenced by a complex adaptive landscape comprising different regional selective pressures, and do not reflect a simple model of dispersal and integration following the introduction of a novel pathogen [35]. But we assume that the emergence of the aggressive pathogen *F. graminearum* on the territory where grain is cultivated implies the emergence of a problem with DON for years to come. It should be noted that the present data expands information about the area of *F. graminearum* in the global space and will get chance to acquainted with peculiarities of population of fungi.

Using the mycological method, the presence of *F. sporotrichioides* was established in 57.9% of the analyzed grain samples, whereas the DNA of this pathogen was found in 84.1% of the grain samples. In West Siberia, *F. sporotrichioides* was occurred in the 73.1% of samples with the average rate of grain infection 3.1%, although the DNA content of the fungus was detected in all the samples and reached 2151 pg/ng. Occurrence of this pathogen in West Siberia was significantly higher than in two others regions. *F. sporotrichioides* infection was detected in 33.3% of the samples from the Volga region and in 61.5% of the samples from the Ural region; the DNA of *F. sporotrichioides* was detected by qPCR in 61.1% and 92.3% of these samples, respectively.

At the same time, the occurrence of *F. sporotrichioides* did not lead to significant contamination of the grain with the mycotoxins and were not found the differences in amounts in the regions. T-2 toxin was detected in three of the samples analyzed by ELISA and in 12 samples using the HPLC-MS/MS method, with a content of less than 100 ppb. However, the analysis of its derivate HT-2 toxin using the chromatographic method led to its detection in 38 samples. This mycotoxin was detected in 84.5% of the grain samples from West Siberia, with a content of less than 100 ppb. In the Volga region, HT-2 toxin was found in 38.9% of the samples, and in the Ural region, it was found in 61.5% of the samples. In four of these samples (Bashkortostan, Tatarstan, and Chelyabinsk Oblast), the HT-2 toxin content was exceeded 100 ppb. These regions are notorious for an outbreak of a mycotoxin-induced disease in the 1930–40s known as alimentary toxic aleukia, which led to the deaths of thousands of people and animals [36]. Subsequently, the disease was determined to be caused by T-2 toxin produced by *F. sporotrichioides* growing on grains overwintered in the fields.

At a later date, when grain from West Siberia was analyzed with ELISA, the researchers noted the wide occurrence of this pathogen and a significant T-2-toxin content [37,38]. In all likelihood, the environmental conditions of this region contribute to the existence and spread of *F. sporotrichioides*. Indeed, a study of batches of wheat, barley, and oats harvested in 1995–2001 in the Ural region and in West Siberia using ELISA found T-2 toxin, on average, in 36.3% (5.1–69.4%) and 37.7% (5.9–86.7%) of the grain samples, respectively [38]. However, high levels of contamination exceeding the maximum allowable level were found in 2–3% of the analyzed samples in each region.

The detection of significantly high amounts of HT-2 toxin in comparison to T-2 toxin in this study was unexpected. In the Russian Federation, a maximum permissible level has been established for T-2 toxin, and therefore mass screening of grain for this metabolite is carried out; however, HT-2 toxin is not usually investigated. This likely lead to an underestimation of the risk of grain contamination by *F. sporotrichioides* toxins. Our results highlight the need to consider the co-presence of T-2 and HT-2 toxins in grain.

Interestingly, a high correlation was found between the *F. graminearum* and *F. sporotrichioides* DNA contents in the grain, which also led to a significant correlation of DON and ZEN contents not only with the DNA of their producer *F. graminearum* but also with *F. sporotrichioides* DNA. One of the explanations for this phenomenon could be the similar susceptibility of cultivated wheat varieties, which, under favorable conditions for *Fusarium* infection, are not able to resist the penetration of all pathogenic species and thus the formation of mycotoxins.

The well-known producers of T-2 and HT-2 toxins are *F. sporotrichioides*, *F. langsethiae*, and *F. sibiricum* Gagkaeva, Burkin, Kononenko, Gavrilova, O’Donnell, and Aoki and Yli-Mattila [39,40]. In this study, we observed only *F. sporotrichioides*, although *F. sibiricum* previously has been detected in Siberia and the Russian Far East [40], and one strain of *F. langsethiae* has been found on Asian territory to date (the Ural region, Tyumen Oblast) [41]. The growth rate and biomass of aerial mycelium of *F. langsethiae* are significantly lower than those of *F. sporotrichioides*, so it is possible that during mycological analysis the colonies of this species were unnoticed under other fast-growing fungal colonies. Although we can only speculate, it appears that *F. langsethiae* and *F. sibiricum* may contribute to the contamination of grain with T-2 and HT-2 toxins in the Ural and the West Siberian regions. This opinion could be confirmed by the absence of high correlation between the amount of these mycotoxins and grain infection, *F. sporotrichioides* DNA content, and the results of all relevant immunoassays.

The observed often lack of relationship between the grain infection and the biomass of mycotoxins producer is quite an explicable. Depending on the time of infection, environmental conditions, and the susceptibility of the cultivar, the depth of the grain infection can vary significantly, as filaments of fungi can present on or in the surface layers or fully occupy the internal tissues of the endosperm [42,43].

In general, this study shows a need for further research on the *Fusarium* species capable of producing trichothecenes type A as well as the factors that affect mycotoxin production in grains, even though considerable progress has already been made in this area [44,45,46,47,48].

Development of accurate, rapid, specific, and easy protocols for early detection of the critical toxigenic *Fusarium* species will open up broad new fields of applications that could lead to improvements in healthy cereal production. Strict control of the contamination of grains used for food and feed is crucial for reducing the human and animal health risks related to mycotoxins.

## 4. Conclusions

For the first time, the *Fusarium* infection of wheat grain samples obtained from the Volga, Ural, and West Siberia regions of Russia were analyzed using highly sensitive analytical methods. Regional differences were identified regarding the contamination of wheat grain by *Fusarium* fungi and their toxins. The presence of *F. graminearum* and its toxic metabolites, such as DON and ZEN, in the Ural and West Siberia regions was confirmed. According to the ELISA and HPLC-MS/MS results, the DON content in wheat grain samples from West Siberia were 3–4 times higher than the permissible level. Simultaneously, the wide occurrence of *F. sporotrichioides* in the analyzed samples did not lead to significant contamination of the grain with the mycotoxins produced by this fungus. In addition, significantly higher levels of HT-2 toxin in comparison with T-2 toxin were found in the samples.

## 5. Materials and Methods

### 5.1. Wheat Grain Samples

In total, 57 wheat grain samples were collected from three regions of Russia (Volga, Ural, and West Siberia) and used for laboratory analyses (Table 4, Table S1). The Volga region (18 samples) was represented by Orenburg Oblast, Bashkortostan as well as Penza Oblast, Volgograd Oblast, Samara Oblast, and Tatarstan. The samples from the Ural region (13 samples) were collected in Chelyabinsk Oblast and Tyumen Oblast. The remaining 26 grain samples were collected in the West Siberian region: Altay Krai, Krasnoyarsk Krai, Novosibirsk Oblast, and Omsk Oblast.

All of the grain samples were harvested from commercial fields in 2017 and represented the different varieties of wheat recommended for cultivation in these regions (the effect of variety has not been taken into account in this study).

### 5.2. Isolation and Morphological Characterization of *Fusarium spp.* in Wheat Grain

The 100–150 grains of each sample were surface sterilized with a 5% sodium hypochlorite solution for 1–3 min. Then, the grains were washed with sterile water and put into Petri dishes on self-made potato sucrose agar medium (PSA) containing 1 mL/L of a mixture of antibiotics (HyClone ™, GE Healthcare Life Sciences, Wien, Austria), which suppressed the growth of bacteria, and 0.4 μL/L of Triton X-100 solution (Panreac, Barcelona, Spain), which reduced the linear growth of mycelial fungi. After 7 days of incubation in the dark at 24 °C, the number and the species composition of the fungi were counted and identified. The taxonomic status of isolated fungi was determined according to the sum of their morphological features [49]. The grain infection by the principal toxigenic *Fusarium* species (*F. graminearum* and *F. sporotrichioides*) was calculated as the ratio of the number of grains from which these fungi were isolated to the total number of analyzed grains and expressed as the incidence percentage.

### 5.3. Sample Preparation for Molecular and Biochemical Analysis

All of the grain samples (20 g) were homogenized separately in sterilized grinding chambers of a batch mill Tube Mill Control (IKA, Königswinter, Germany). The speed of grain grinding was 20,000 rpm for 1 min. The ground wheat flour was stored at –20 °C until further analyses.

### 5.4. DNA Extraction and Quantitative PCR

The total DNA from 200 mg of wheat flour was isolated using the Genomic DNA Purification Kit (Thermo Fisher Scientific, Vilnus, Lithuania) based on a modification of the manufacturer’s protocol. Using the same kit, DNA was also isolated from the mycelium of typical strains of *F. graminearum* and *F. sporotrichioides* cultivated on PSA. All the typical strains of fungi are maintained in the Collection of the Laboratory of Mycology and Phytopathology of the All-Russian Institute of Plant Protection (St. Petersburg, Russia).

The quality of the fungal DNA was checked with the primer pair ITS1/ITS4 [50]. DNA concentrations from the grain samples and fungal isolates were determined using a Qubit 2.0 Fluorometer with a Quant-iT dsDNA HS Assay Kit (Thermo Fisher Scientific, Waltham, MA, USA). Before the start of qPCR, the concentrations of all DNA samples were aligned to 20–85 ng/μL.

The amount of *F. graminearum* and *F. sporotrichioides* DNA was determined in every total DNA sample. The DNA content of the *F. graminearum* was evaluated by qPCR with TaqMan probes [13]. The reaction was carried out in a 20 μL volume containing 10 μL of a 2 × TaqM master mix (AlkorBio, St.Petersburg, Russia), 300 nM of each primer, 100 nM of a fluorescent sample (Evrogen, Moscow, Russia), and 2 μL of the corresponding DNA solution.

The DNA content of *F. sporotrichioides* was determined using qPCR with SYBR Green dye [13]. The reaction was carried out in a 20 μL volume containing 4 μL of a 5 × qPCRmix-HS SYBR master mix (Evrogen, Moscow, Russia), 500 nM of each primer, and 2 μL of the DNA solution.

All qPCR assays were run using the CFX 96 Real-Time System thermocycler (BioRad, Hercules, CA, USA). The DNA solutions of the *Fusarium* strains were diluted to 10 ng/μL and used to construct calibration curves in subsequent dilutions of factors of 10 from 10^–1^ to 10^–6^ ng/μL. Fold-differences and standard errors were calculated from the Ct values, which were normalized against the DNA of pure cultures of *F. graminearum* and *F. sporotrichioides* using the Bio-Rad CFX Manager 1.6 software package. DNA content was presented as the ratio of fungal DNA to total DNA in each sample (pg/ng). The low quantification limit of 5 × 10^−4^ pg fungal DNA on one ng of total DNA was established as the threshold value of DNA in a sample, which can be quantitatively determined with high precision. All samples were analyzed at least twice.

### 5.5. Immunoassays for Detection of *Fusarium* Biomass

The extraction was carried out from 1 g of wheat flour by extensive mixing with 0.1 M phosphate-buffered saline (pH 7.2–7.4) at 1:10 (*w*/*v*) on the shaker platform ST-3L (ELMI, Riga, Latvia) for 30 min at ambient temperature (+20–25 °C). Then, the extracts were cleared by centrifugation for 15 min at 3000× *g* at +2–8 °C (GPR centrifuge, Beckman, USA). The obtained supernatants were analyzed immediately or stored at –20 °C until further analyses. In the case of testing a frozen extract, it was first centrifuged following the same procedure, and then the secondary supernatant was used for the analysis.

In the EIA competition format (XEMA, cat# K827L, Moscow, Russia), the flour extract was incubated in a microwell coated with the antigen (fungal extract) along with the specific affinity-purified rabbit polyclonal antibodies. After washing the unbound material, the remaining antibodies were detected using an anti-rabbit-horseradish peroxidase conjugate tracer. The chromogenic reaction signal is inversely related to the concentration of fungal antigens in the sample. The EIA required undiluted grain extracts and was quantitated in units/mL using calibration samples supplied with the assay kits.

### 5.6. Mycotoxin Analysis

ELISA kits of XEMA Co. Ltd. (Moscow, Russia) were used for the analysis of DON (cat. No. K925), ZEN (cat. No. K923) and T-2 toxin (cat. No. K922). Mycotoxin extraction and next-sample pretreatment for each immunoassay kit were performed according to the manufacturer’s instructions. The absorbance of the final solutions was measured with a spectrophotometer (Multiskan EX, Thermo Electron Corporation, Vantaa, Finland) at 450 nm.

HPLC-MS/MS system-tandem liquid chromatography-mass spectrometry was used for the validation of the immunoassay methods (Agilent Infinity LC Systems 1290 and AB SCIEX Triple Quad ™ 5500). The analysis of the mycotoxins was carried out in the laboratory of the All-Russian Scientific and Technological Institute of Poultry (Moscow region), with the support of the Russian branch of the company Biomin, following the described procedure [51]. From every sample 5 g of wheat flour was extracted with 20 mL of extraction solvent (acetonitrile/water/acetic acid 79:20:1, *v*/*v*/*v*). Detection and quantification were performed with a QTrap 5500MS/MS system (Applied Biosystems, Foster City, CA) equipped with a TurboV electrospray ionization (ESI) source and a 1290 series UHPLC system (Agilent Technologies, Waldbronn, Germany). Chromatographic separation was performed at 25 °C on a Gemini^®^C18-column, 150 × 4.6 mm i.d., with a 5 µm particle size, equipped with a C18 security guard cartridge, 4 × 3 mm i.d. (all from Phenomenex, Torrance, CA, US). Elution was carried out in binary gradient mode. Both mobile phases contained 5 mM ammonium acetate and were composed of methanol/water/acetic acid ratios of 10:89:1 (*v*/*v*/*v*; eluent A) and 97:2:1 (*v*/*v*/*v*; eluent B), respectively.

The limit of detection (LOD) determined for each mycotoxin was 4.9, 0.8, 3.25 and 2.7 ppb for DON, ZEN, T-2, and HT-2 toxins, respectively. Concerning the limits of quantification (LOQ), they varied in case of DON in range of 5.8–215.2 ppb, ZEN—1.1-40.8 ppb, T-2 toxin—5.23–193.6 ppb, and HT-2 toxin—3.48–129.2 ppb.

Regardless of the method of mycotoxin analysis, the extracts exceeding the upper limits of mycotoxin detection were diluted and reanalyzed, and the dilution factor has been used for calculations of the concentrations of mycotoxins. All samples were analyzed at least twice.

### 5.7. Statistical Analysis

Data were analyzed using Microsoft Office Excel 2010 (Microsoft, Redmond, WA, USA) and Statistica 10.0 (StatSoft, Tulsa, OK, USA). To center the distribution of quantitative parameters, in addition to the mean, the median was also calculated. Since the original data did not assume normal distribution, the Mann-Whitney U test, non-parametric alternative of t-test, was used to test whether the medians of samples are different (*p* ≤ 0.05) [52]. The relationship between the quantitative traits was evaluated using the linear Pearson correlation coefficient (*r*) at a significance level of *p* ≤ 0.01.

## Figures and Tables

**Table 1:** Infection of the wheat grains by *Fusarium* fungi.

Parameters		Origin of Wheat Grain (the Region and Numbers of Samples)		
		Volga (*n* = 18)	Ural (*n* = 13)	West Siberia (*n* = 26)
Samples infected by *Fusarium* fungi, %		72.2	69.2	96.1
FDG on average/median (range), %		3.3/1.5 a (0–12)	5.2/2.0 a (0–30)	11.3/7.0 b (0–38)
The incidence of *F. graminearum* on average/median (range), %		0 a	0.1/0 ab (0–1)	0.8/0 b (0–10)
The incidence of *F. sporotrichioides* on average/median (range), %		0.8/0 a (0–6)	2.2/ 1.0 ab (0–9)	3.1/2.5 b (0–15)
The amount of *Fusarium* DNA×10^−4^ on average/median (range), pg/ng	*F. graminearum*	0 a	55/0 b (0–593)	1483/0 c (0–1318)
	*F. sporotrichioides*	1141/207 a (0–4458)	1192/1292 ab (0–3793)	2151/1445 b (167–6740)
The amount of *Fusarium* antigens on average/median (range), U/mL		23.9/13.7 a (2–117)	25.6/18.0 a (1–95)	197.7/39.0 b (8–1820)

Medians indicated by the same letter in row are not statistically significantly different (Mann-Whitney U test; *p* ≤ 0.05).

**Table 2:** *Fusarium* mycotoxins content in the wheat grains from different regions estimated by ELISA and HPLC-MS/MS.

Method	Mycotoxins	Amount of Mycotoxins on Average/Median (Range), ppb		
		Volga (*n* = 18)	Ural (*n* = 13)	West Siberia (*n* = 26)
ELISA	DON	5.5/0 a (0–100)	11.5/0 a (0–150)	224.3/0 a (0–2787)
	ZEN	0 a	0 a	2.6/0 a (0–21)
	T-2 toxin	3.4/0 a (0–40)	5.7/0 a (0–74)	0.9/0 a (0–22)
HPLC-MS/MS	DON	5.3/0 a (0–74)	36.1/0 ab (0–415)	283.1/0 b (0–2239)
	ZEN	0 a	0 a	8.5/0 a (0–106)
	T-2 toxin	3.4/0 a (0–29)	7.2/0 a (0–66)	2.5/0 a (0–17)
	HT-2 toxin	25.9/0 a (0–170)	36.2/15.4 a (0–152)	31.0/11.5 a (0–92)

Medians indicated by the same letter in row are not statistically significantly different (Mann-Whitney U test; *p* ≤ 0.05).

**Table 3:** The relationships between the parameters of wheat grain contamination by *Fusarium* fungi and mycotoxins estimated using various methods.

Estimated Parameters			Infected Grain Measured by Mycological Method			Amount of DNA of *Fusarium* Fungi Measured by qPCR		Amount of Antigens of *Fusarium* Fungi Measured by EIA	Amount of Mycotoxins of *Fusarium* Fungi Measured by			
			*F. gram.* ^1^	*F. spor.*	FDG				ELISA		HPLC-MS/MS	
						*F. gram.*	*F. spor.*		DON	T-2	DON	T-2
Infected grain measured by mycological method		*F. gram.*	1.0									
		*F. spor.*	0.24									
		FDG	0.43 *	0.70 *								
Amount of fungal DNA measured by qPCR		*F. gram.*	0.42 *	−0.07	0.10							
		*F. spor.*	0.30	0.43 *	0.38 *	0.54 *						
Amount of antigens of *Fusarium* fungi measured by EIA			0.38 *	−0.08	0.13	0.98 *	0.56 *					
Amount of mycotoxins of *Fusarium* measured by	ELISA	DON	0.30	−0.15	0.07	0.94 *	0.53 *	0.97 *				
		T-2 toxin	−0.06	−0.05	−0.04	0.10	0.22	0.12	0.15			
	HPLC-MS/MS	DON	0.45 *	−0.04	0.13	0.99 *	0.55 *	0.96 *	0.93 *	0.09		
		T-2 toxin	0.12	0.14	0.10	0.06	0.33	0.06	0.03	0.03	0.07	
		НТ-2 toxin	0.11	0.24	0.13	0.06	0.57 *	0.07	0.03	0.03	0.07	0.78 *

^1^*F. gram.*—*F. graminearum*, *F. spor.*—*F. sporotrichioides*, FDG—*Fusarium* damaged grain; * Correlation coefficients are significant at *p* ≤ 0.01.

**Table 4:** Climatic data during the growing season of 2017 in the regions of grain samples origin.

Region (Numbers of Samples)	Month	Average Month Temperature, °C			Average Humidity, %	Average Rainfall, mm
		Mean	Min	Max		
Volga (*n* = 18)	June	20.3	10.4	30.1	56	34.0
	July	21.0	12.4	29.4	58	28.9
	August	21.5	11.9	31.6	56	7.3
Ural (*n* = 13)	June	18.4	8.5	27.6	63	55.0
	July	17.3	8.8	25.6	71	48.5
	August	18.0	8.7	28.1	71	26.8
West Siberia (*n* = 26)	June	18.6	9.1	28.0	60	32.9
	July	19.6	11.8	28.0	71	45.6
	August	17.5	8.8	26.5	70	34.5

## Supplementary Materials

The following are available online at https://www.mdpi.com/2072-6651/11/5/252/s1. Table S1: List of the results obtained with the various analytical methods.
